# Supplementary material for: Steroid hormones sulfatase inactivation extends lifespan and ameliorates age-related diseases
Source: Nat Commun. 2021 Jan 4;12:49. doi: 10.1038/s41467-020-20269-y (PMC7782729; doi:10.1038/s41467-020-20269-y)
Supplement: Supplementary file 1 — Supplementary information [file 41467_2020_20269_MOESM1_ESM.pdf]

SUPPLEMENTARY INFORMATION

Steroid hormones sulfatase inactivation extends lifespan and ameliorates age-related diseases

Pérez-Jiménez et al.

Contents:

- Supplementary Figure 1-10
- Supplementary Tables 1-3

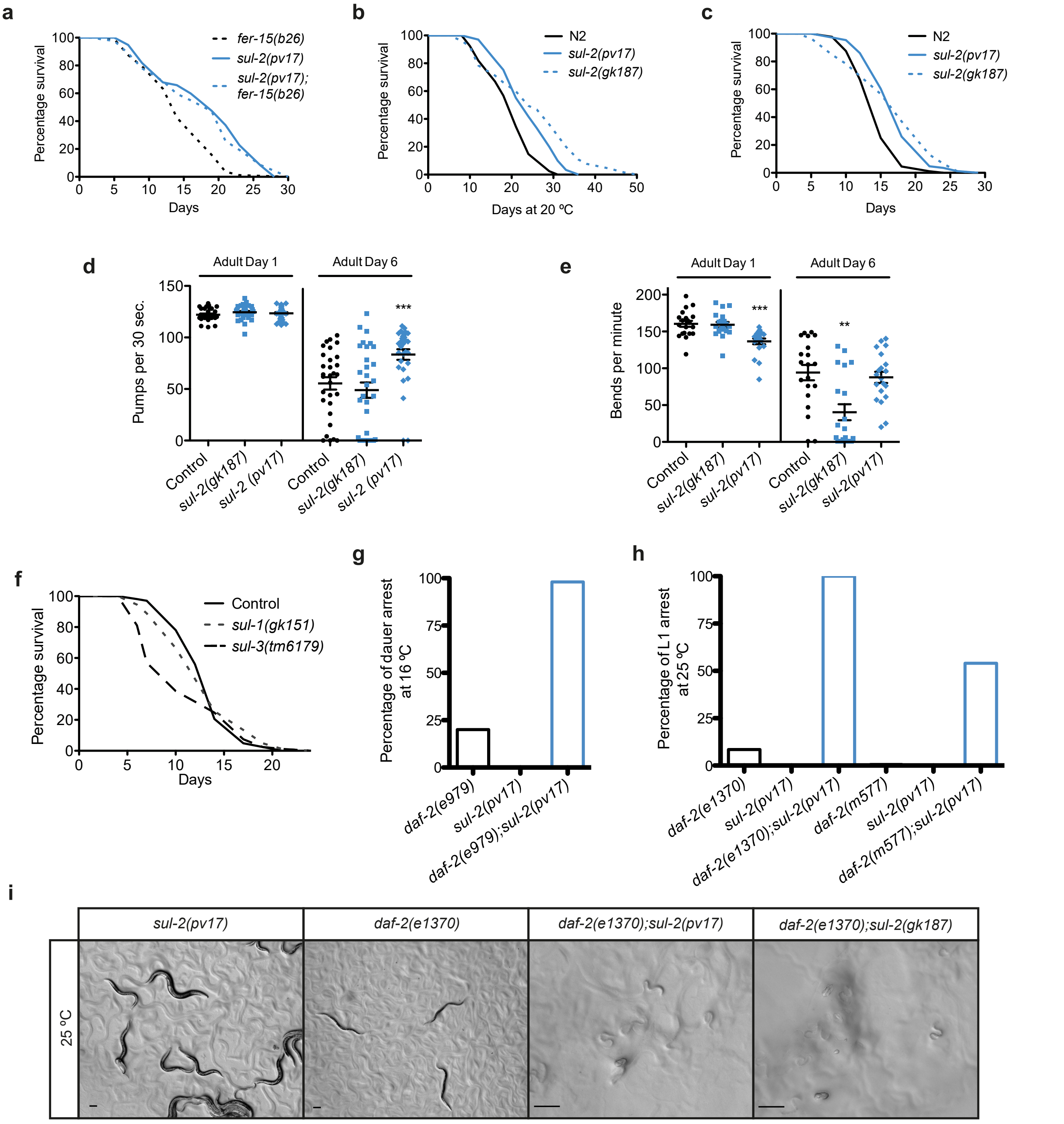

Supplementary Fig. 1 Longevity assays of *sul-2* and genetic interactions with *daf-2*. *sul-2* mutants do not show visible phenotypes, but are long-lived and enhance developmental phenotypes of *daf-2* mutants. **a** *sul-2(pv17)* is long-lived and *fer-15(b26)* does not affect its lifespan. **b** *sul-2* mutants are long-lived at 20 ºC, **c** and at 25 ºC. **d** Pumping rate of *sul-2* mutants are similar to wild type at adult day 1, while *pv17* allele keeps higher pumping rate than wild type at adult day 6 stage. Data display from two independent biological assays; n=30 *per* day. Two-tailed Mann-Whitney t-test. **e** Thrashing in *sul-2(gk187)* is similar to wild type, but is slightly lower in *sul-2 (pv17)* at 25 ºC on day 1 of adulthood. On day 6 of adulthood at 25 ºC, **d** the trashing on *sul-2(gk187)* is lower than wild type and *sul-2(pv17),* with no differences among these last strains. The population of *sul-2(gk187)* at day 6 seems to distribute into two different populations, one with almost no thrashing and other with values similar to wild type and *sul-2(pv17)*. Data displayed from two independent biological assays; n=20 *per* day. Two-tailed Mann-Whitney t-test. **f** *sul-1(gk151)* and *sul-3(tm6179)* do not increase life span. **g** A small percentage of *daf-2(e979)* animals arrest development in dauer larvae at 16 ºC, *sul-2(pv17)* mutant does not show any larval arrest, but enhances dauer arrest of *daf-2(e979)*. **h** Most *daf-2(e1370)* animals arrest in dauer stage when develop at 25 ºC, but small percentage arrest in L1 stage. In this condition, all animals from *daf-2(e1370);sul-2(pv17)* double mutants arrest at L1 stage. Similarly, more than 50% of animals arrest in L1 stage in *daf-2(m577);sul-2(pv17)* background, while none of the single mutants show this phenotype. **i** Example of *sul-2(pv17)* larvae (similar to *sul-2(gk187),* image not shown), dauer larval arrest of *daf-2(e1370)* or L1 arrest of *daf-2(e1370);sul-2(pv17)* and *daf-2(e1370);sul-2(gk187)* at 25 ºC. Statistics and additional longevity curves are shown in Supplementary Dataset 1. In **d** and **e** Mean±SEM are displayed. **p≤0.01, ***p≤0.001. Scale bars represent 100 µm. Source data are provided as a Source Data file.

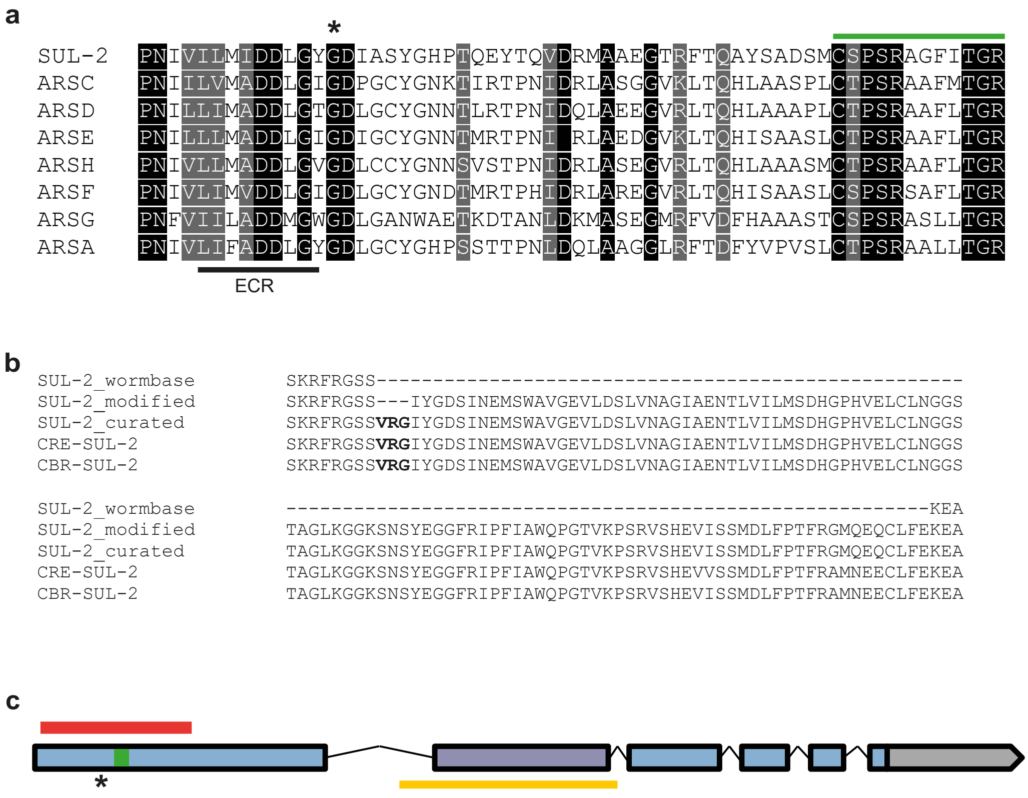

Supplementary Fig. 2 Identification of *pv17* allele and curated sequence of SUL-2. a The *pv17* allele is a missense mutation that changes the glycine indicated with asterisk to an aspartic acid residue. The mutation is located close to an evolutionary constrained region (ECR)[1], indicated with the bar at the bottom and also near to the catalytic core of sulfatases (in green). **b** The DNA sequence we identified in the wild type matches the one publish in GeneBank (LK927547.1) which differs to the first published in wormbase identified as orthologue to ARSA (https://wormbase.org/species/c_elegans/-gene/WBGene00006309#0-9f-10, Section: Legacy manual gene description), that misses 459 bp. The sequence of the cDNA clone yK387h10.5 (genbank MW145131) identified a new exon in that gap. This new exon was also predicted by Li et al. (2015)[2] based on RNAseq data, but such prediction missed three aminoacids (in bold). Notice that the three aminoacids we identified in this sequence are present also in other species (CRE: C. remanei, CBR: C. brigssae). **c** Exon intron composition of wild type *sul-2* and region deleted in the *gk187* allele (in red). The mutant lesion is available at wormbase (https://wormbase.org/species/c_elegans/variation/WBVar00145594#02-456-10). This allele deletes the sequence that encodes to the catalytic core of sulfatases (in green) and generates a frame shift, conserving only the four first aminoacids of the original sequence; therefore we considere *gk187* a null allele. The *pv17* allele location is indicated with asterisk, the new DNA fragment identified in yellow and the new exon in purple.

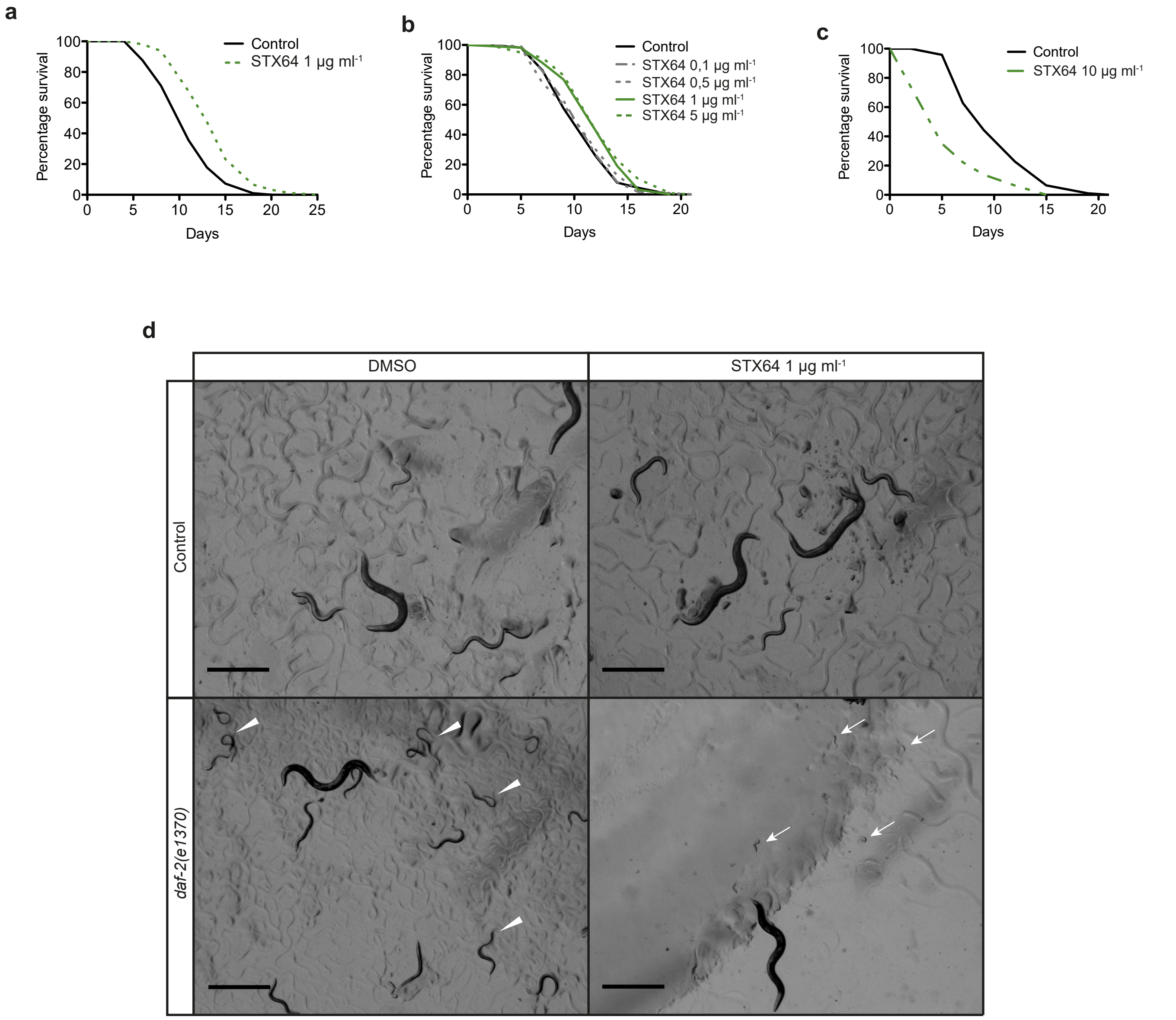

Supplementary Fig. 3 Treatment with STX64 phenocopies *sul-2* mutants. a STX64 in non-UV *E. coli* increases lifespan of wild type. **b, c** Dosis curves of STX64 in non-UV *E.coli* show a significat effect at 1 µg ml^{-1} and 5 µg ml^{-1} **d** Photographs of wild type and *daf-2(e1370)* at 25 °C. DMSO (STX64 vehicle) does not affect development of wild type, neither STX64 treatment, *daf-2(e1370)* arrests mostly in dauer stage at 25 °C, but mainly arrests in L1 when treated with STX64, similar interaction is also observed in the *sul-2* mutants. Photographs were taken in a Leica scope. Arrow heads: dauers, arrows: L1s. Scales bar represent 500 µm. Statistics and additional longevity curves are shown in Supplementary Dataset 1.

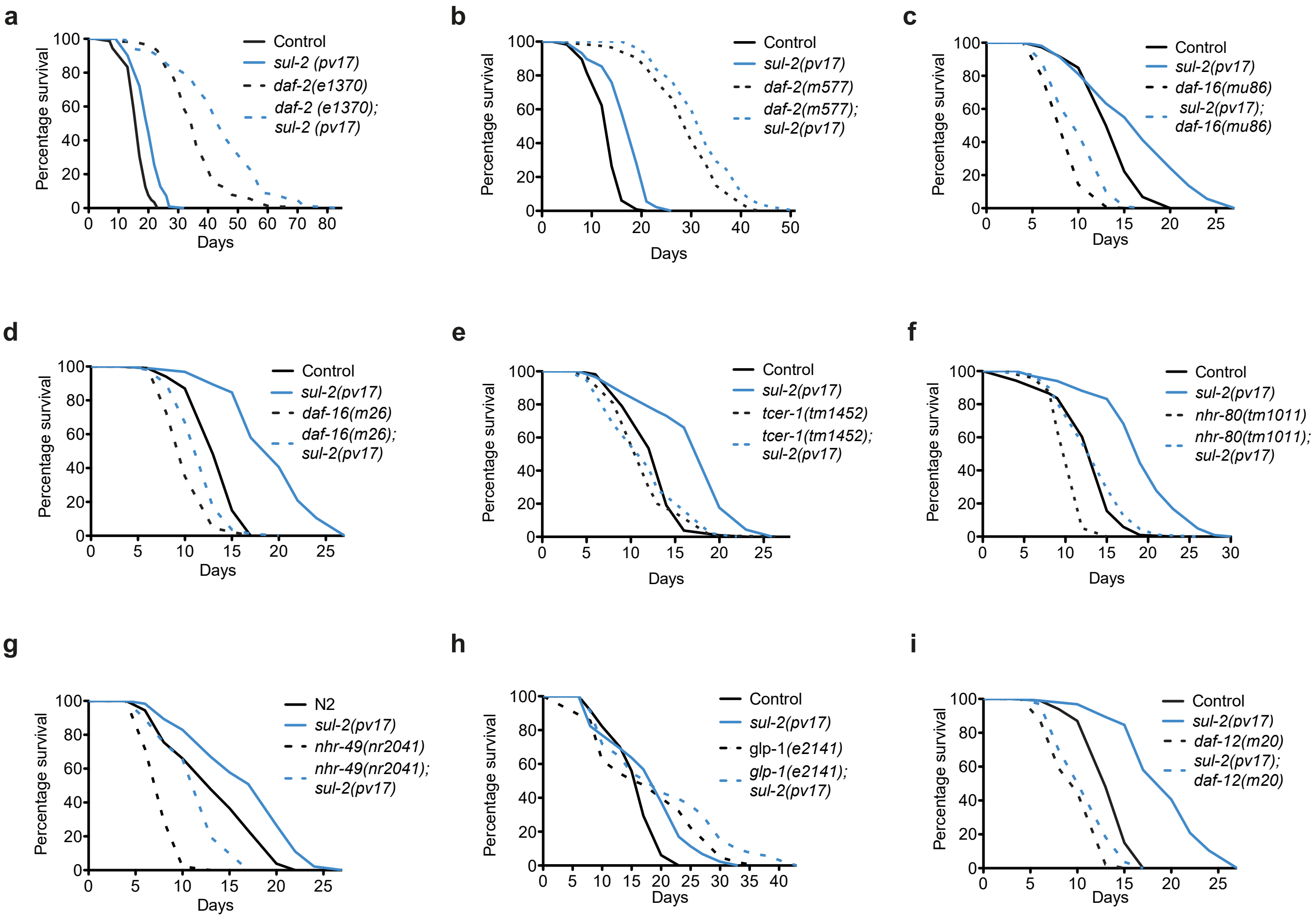

Supplementary Fig. 4 Genetic interactions of *sul-2(pv17)* allele. *sul-2* point mutation allele *pv17* shows similar phenotypes in longevity interactions assayed as *sul-2* deletion, except in *glp-1* background. **a, b** *sul-2(pv17)* enhances longevity of two *daf-2* alleles, *e1370* and *m577*. **c, d** *sul-2(pv17)* longevity is suppresed by two alleles of *daf-16*, *mu86* and *m26*. **e** *sul-2(pv17)* longevity is suppresed by *tcer-1(tm1452)*. **f** *sul-2(pv17)* longevity is partially suppresed by *nhr-80(tm1011)*. **g** *sul-2(pv17)* longevity is not suppresed by *nhr-49(nr2041)*. **h** *sul-2(pv17)* increases lifespan in *glp-1* background. **i** *sul-2(pv17)* longevity is suppresed by *daf-12(m20)*. Statistics and additional longevity curves are shown in Supplementary Dataset 1.

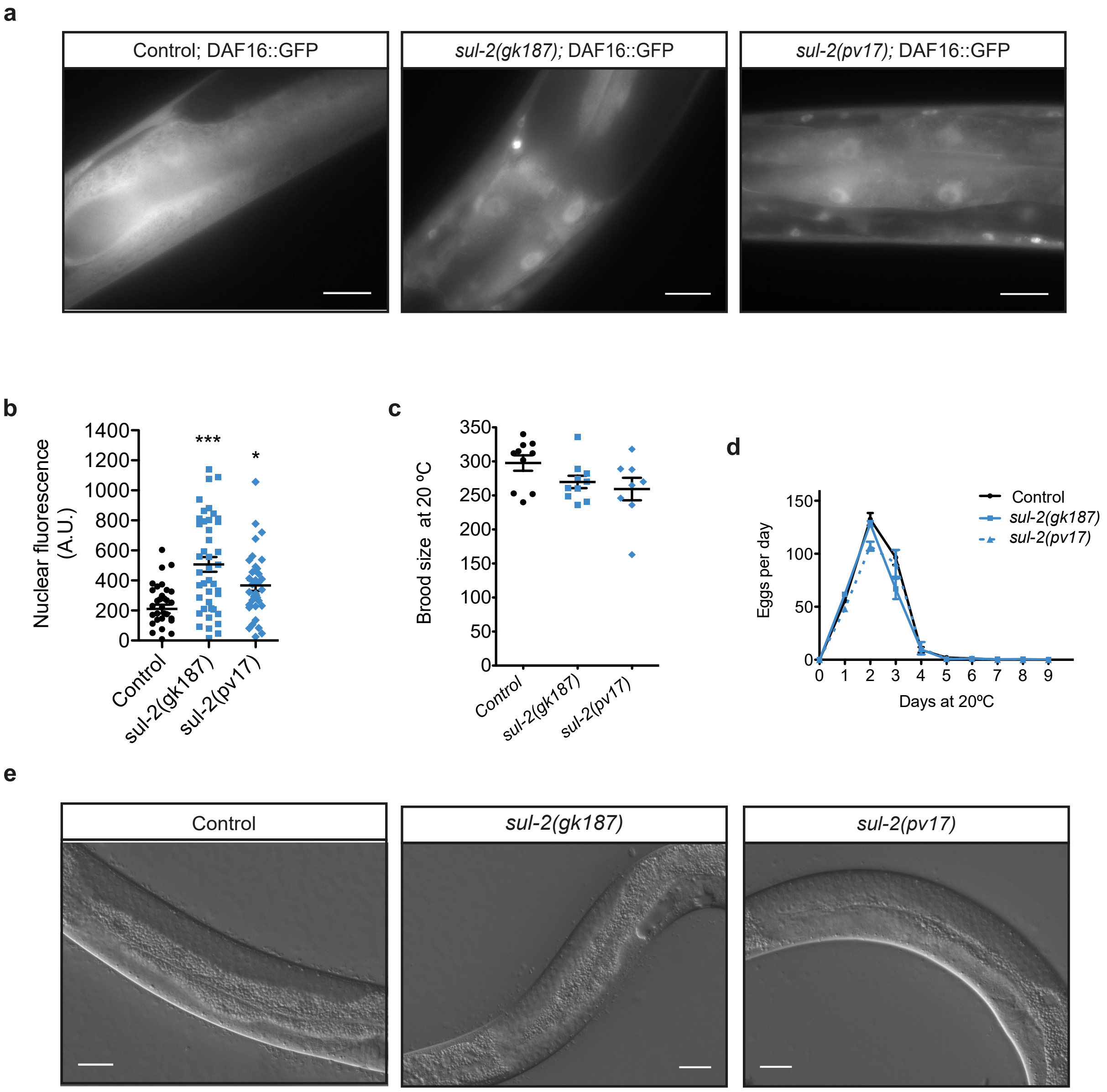

Supplementary Fig. 5 *sul-2* mutants affect DAF-16 location but not reproduction. a Micrographs show representative images of *Pdaf-16::gfp::daf-16* location in wild type (left panel) and *sul-2* mutants (central and right panels). Both *sul-2* mutants increase the nuclear location of DAF-16 in intestinal cells, like in germ-line less animals. Scale bar 20 µm. **b** Quantifications of nuclear fluorescence in the anterior intestinal cells. Data from two independent assays; n≈34 nuclei *per* strain. One-way ANOVA test. **c, d** *sul-2* mutants have similar brood size to control. One-way ANOVA test; ns, and the reproductive period of *sul-2* mutants are not affected. Data from an independent experiment (20 ºC) of two is shown. n≈8 *per* strain. **e** *sul-2* mutants show normal gonad morphology. Micrographs of one representative gonadal arm in late L4 for each strain are shown. Scale bars represent 20 µm. Statistics of longevity curves are shown in Supplementary Dataset 1. In all graphs Mean±SEM are displayed. *$p \leq 0.05$, ***$p \leq 0.001$. Exact n and p-value are included in Source Data file.

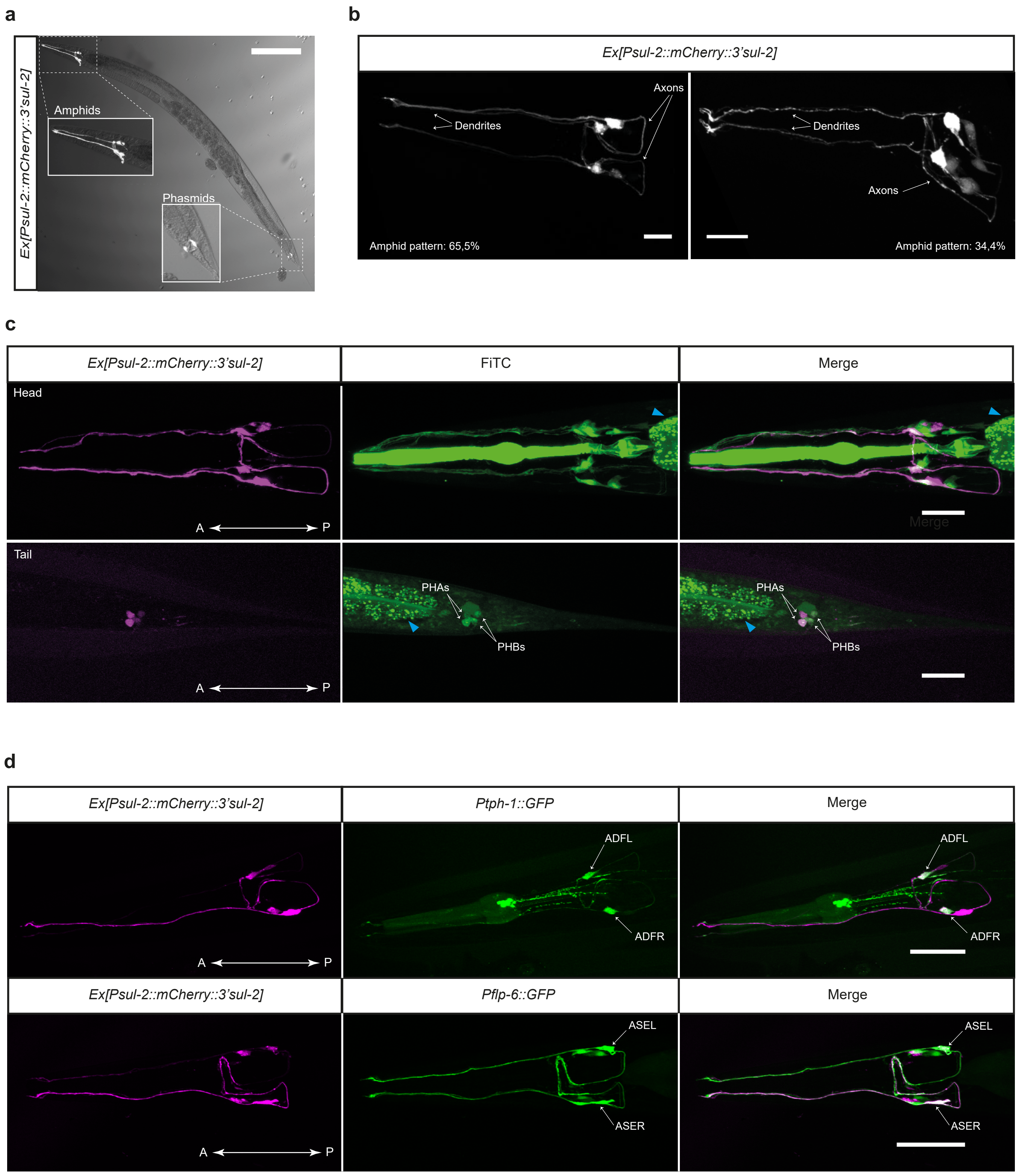

Supplementary Fig. 6 *sul-2* is expressed in amphid and phasmid sensory neurons. Extrachromosomal transgenic worms express mCherry under *sul-2* promoter and its 3'UTR only in few sensory neurons. **a** Transcriptional reporter for *sul-2* is expressed in sensory neurons. Imaged in fluorescence and merged with the bright field. Scale bar represents 200 μm. **b** Representative images of *sul-2* extrachromosomal expression in amphids, most transgenic animals express *sul-2* in two pairs of amphid neurons, left panel, and a portion show expression in other neurons besides of those, right panel. n=64. **c** Collocation of *sul-2* neurons with FiTC stains. Upper panel shows colocalization in the head with the most anterior pair amphids, possibly ASK, ADF or ADL neurons, but not with the most posterior pair, ASG or ASE. In the tail, bottom panel, the four neurons where *sul-2* is expressed colocalize with FiTC staining in PHAs and PHBs phasmids neurons. **d** Identification of the two main pair of amphid where *sul-2* is expressed by collocalization with the *tph-1* neuron-specific promoter for ADF, upper panel, and the posterior neurons expressed by *flp-6* promotor, ASE amphids, bottom panel. Scale bars represent 20 μm in **b, c** and **d**. In the intestine, signals are unspecific from autofluorescence at the conditions imaged (cyan arrow heads).

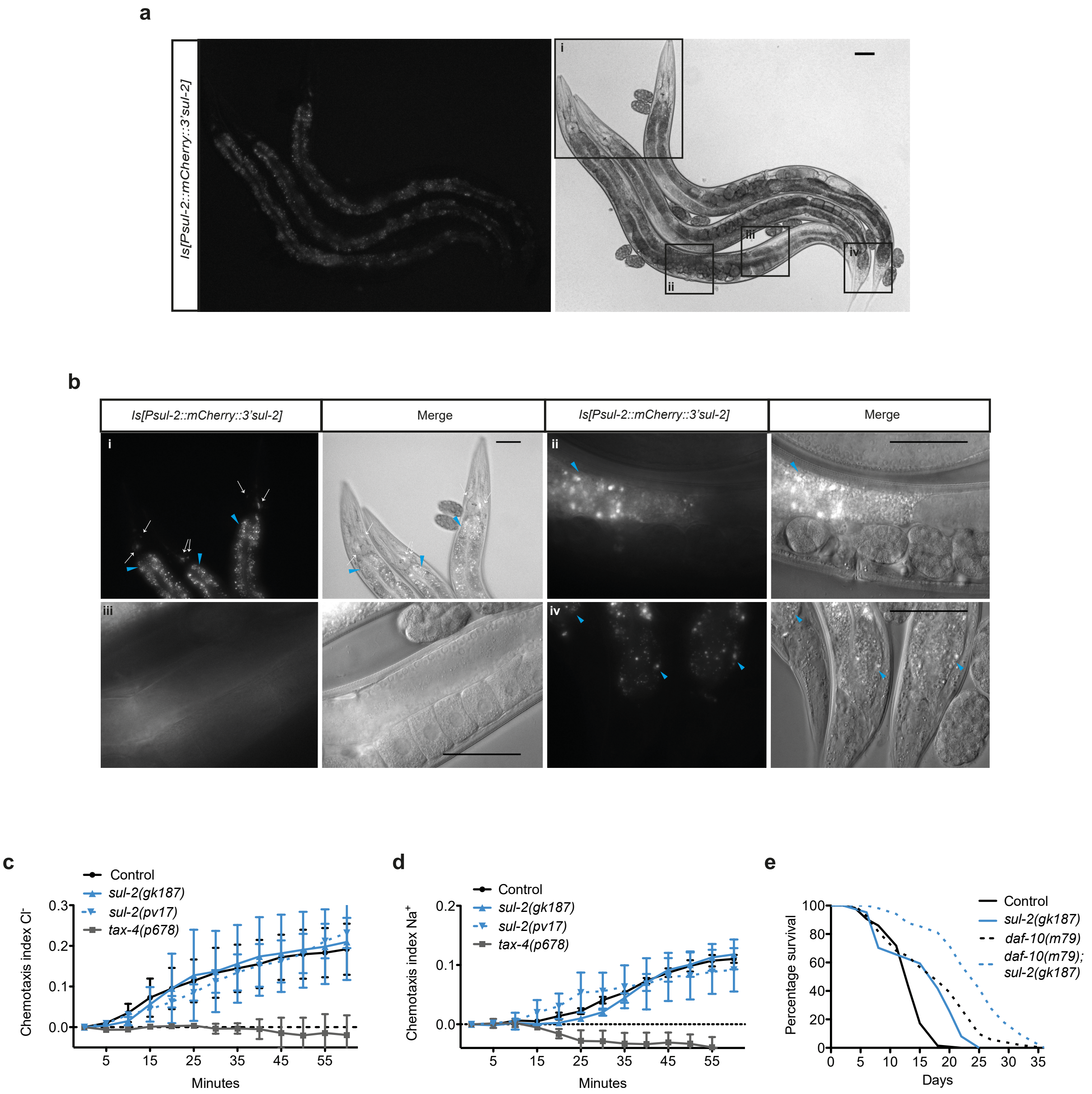

Supplementary Fig. 7 *sul-2* is not expressed in gonadal tissues and is not affected in neuronal functions. a Integrated mCHERRY reporter of the *sul-2* transcriptional unit is only expressed in sensory neurons, there is not expression in other tissues. Image in fluorescence and merged with the bright field image. Scale bar represents 50 μm. **b** Inset of integrated worms, i) In heads, *sul-2* is expressed in few amphid neurons (white arrows). In the intestine, signals are unspecific from autofluorescence at the conditions imaged (cyan arrowheads). ii) There are not specific signals in vulva, embryos or proliferative germline zone. iii) There are not specific signals in gonad or mature oocytes. iv) In the tail, there is not significant signals in phasmids. Scale bars represent 50 μm. **c** Apart from been expressed in the Cl^- and Na^+ sensing neuron (ASE), *sul-2* mutants respond to Cl^- similarly to wild type. *tax-4(p678)* is a negative control. Data from three independent replicates. Mean±SEM are displayed. **d** *sul-2* mutants respond to Na^+ similarly to wild type. *tax-4(p678)* is a negative control. Data from three independent replicates. Mean±SEM are displayed. **e** *sul-2* deletion enhances longevity of the long-lived *daf-10(m79)* mutant, which is affected in sensory neurons. Statistics of longevity curves are shown in Supplementary Dataset 1. Source data are provided as a Source Data file.

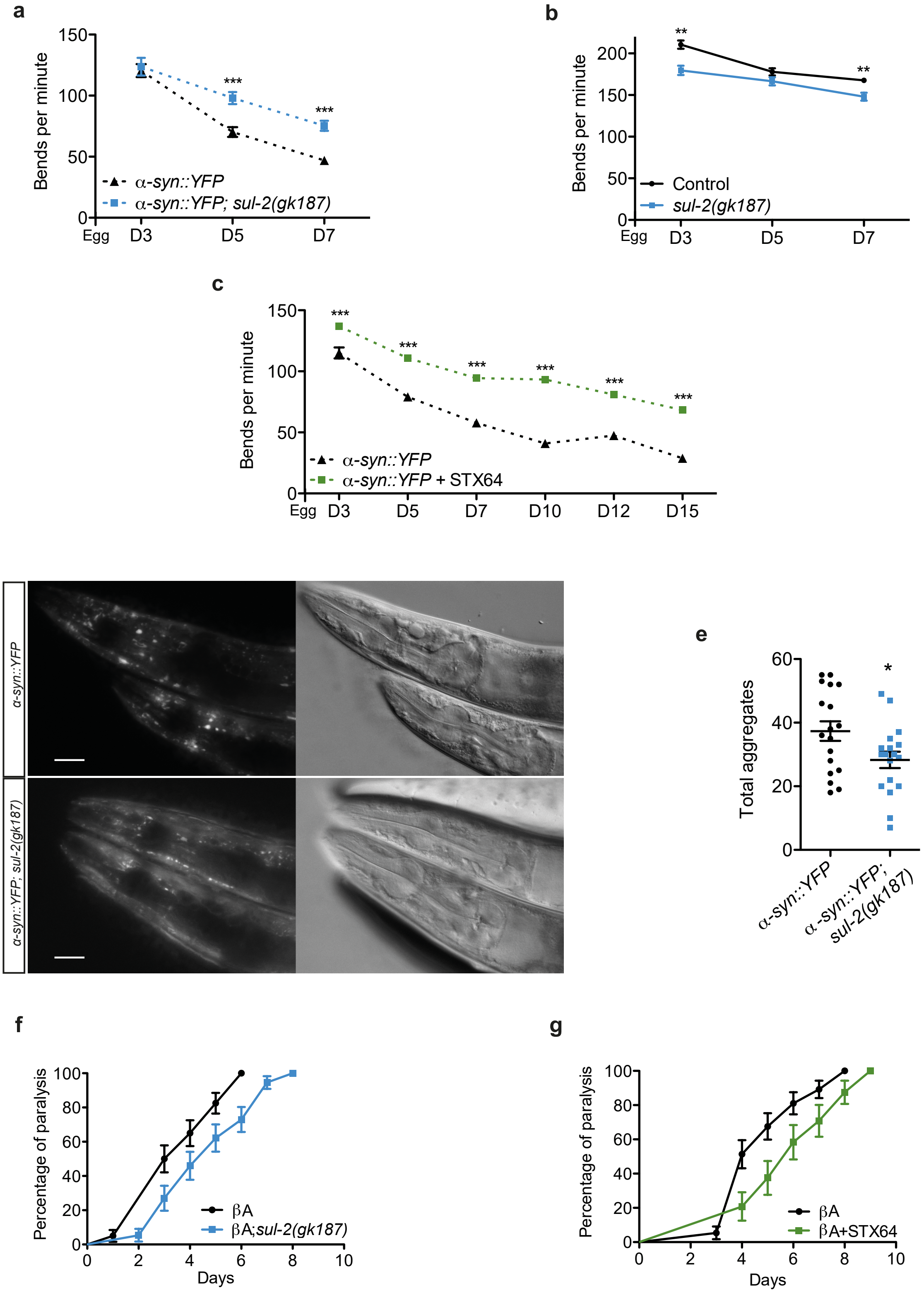

Supplementary Fig. 8 Reduced steroid sulfatase function ameliorates neurodegeneration during aging. a *sul-2* has a beneficial effect during adulthood in muscular Parkinson's disease model. Data display from two independent biological replicates, n≈31 *per* sample, **b** and *sul-2* has less body bends to wild type control at same experimental conditions, n≈15 *per* sample (20 °C). **c** The protective effect of STX64 in muscular Parkinson's disease model is present throughout aging. Data display from two independent biological replicates, n≈12 *per* sample. Two-tailed Mann-Whitney t-test in **a**, **b** and **c**. **d**, **e** *sul-2* reduces significantly the number of α-synuclein aggregates in muscle at 7-day old. Photographs example of animals and quantifications, respectively. n=18. One-tailed unpaired t-test. Scale bars represent 25 μm. In **a**, **b** and **c** Mean±SEM are displayed. *p≤0.05, **p≤0.01, ***p≤0.001. **f** Expression of human β-amyloid in muscle provokes paralysis with age (CL2006 strain) that is ameliorated in *sul-2* deletion mutant. **g** or by STX64 treatment. Data from an independent experiment of two is displayed in **f** and **g**. Log-rank (Mantel-Cox) test, Error bar SE. Curve comparison for *sul-2* deletion vs control p=0.0027 and for STX treatment p=0.007, respectively. Exact n and p-value are included in Source Data file.

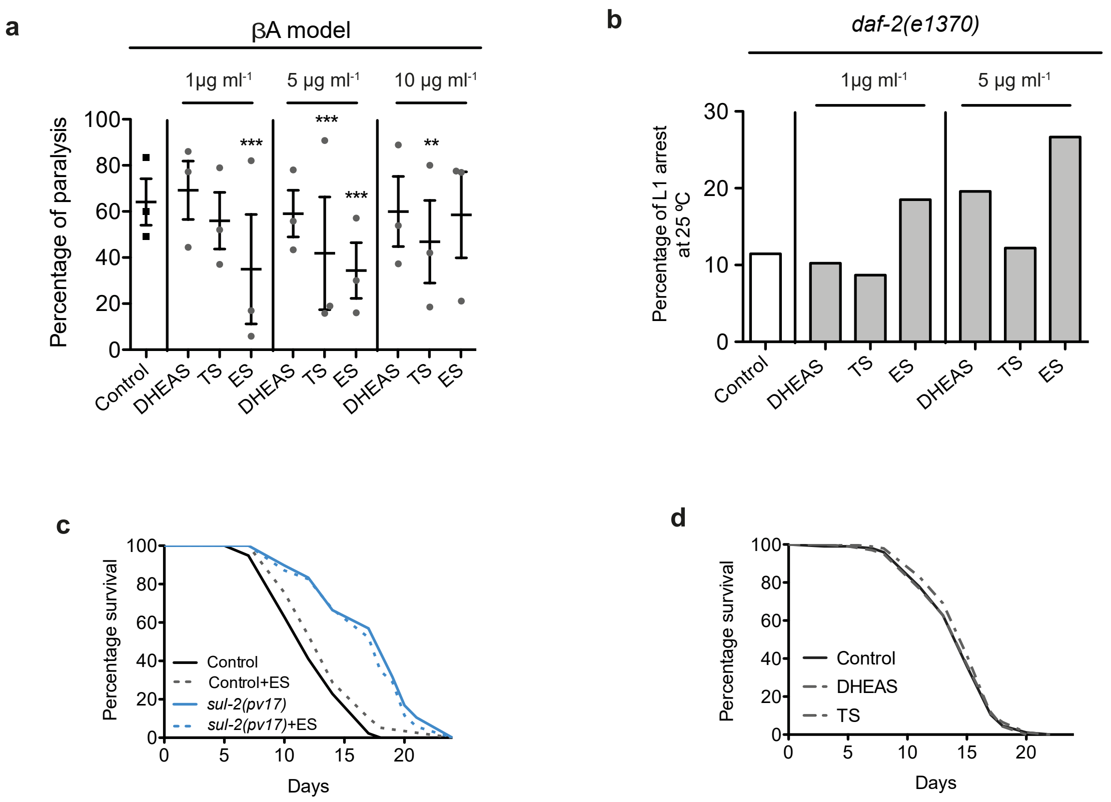

Supplementary Fig. 9 Treatment with sulfated C19 steroid hormones phenocopy *sul-2* inactivation. a Paralysis phenotype of GMC101 strain, Alzheimer's disease model, is reduced with TS and ES, but do not with DHEAS (1μg ml^{-1}). Assays performed with regular NGM plates. Data display the percentages from three independent biological replicates, n≈130 *per* sample. Mean±SEM. χ^2 test; **p≤0.01, ***p≤0.001. **b** Similar to *daf-2(e1370);sul-2*, percentage of L1 arrest in *daf-2(e1370)* increase with DHEAS,TS or ES. **c**, **d** Aditional biological replicates of longevity curve with sulfated C19 steroid hormones. ES (1μg ml^{-1}) increases lifespan in wild type background but does not increase further in *sul-2(pv17)*, while DHEAS and TS do not affect (1μg ml^{-1}). Statistics of longevity curves are shown in Supplementary Dataset 1. Exact n and p-value are included in Source Data file.

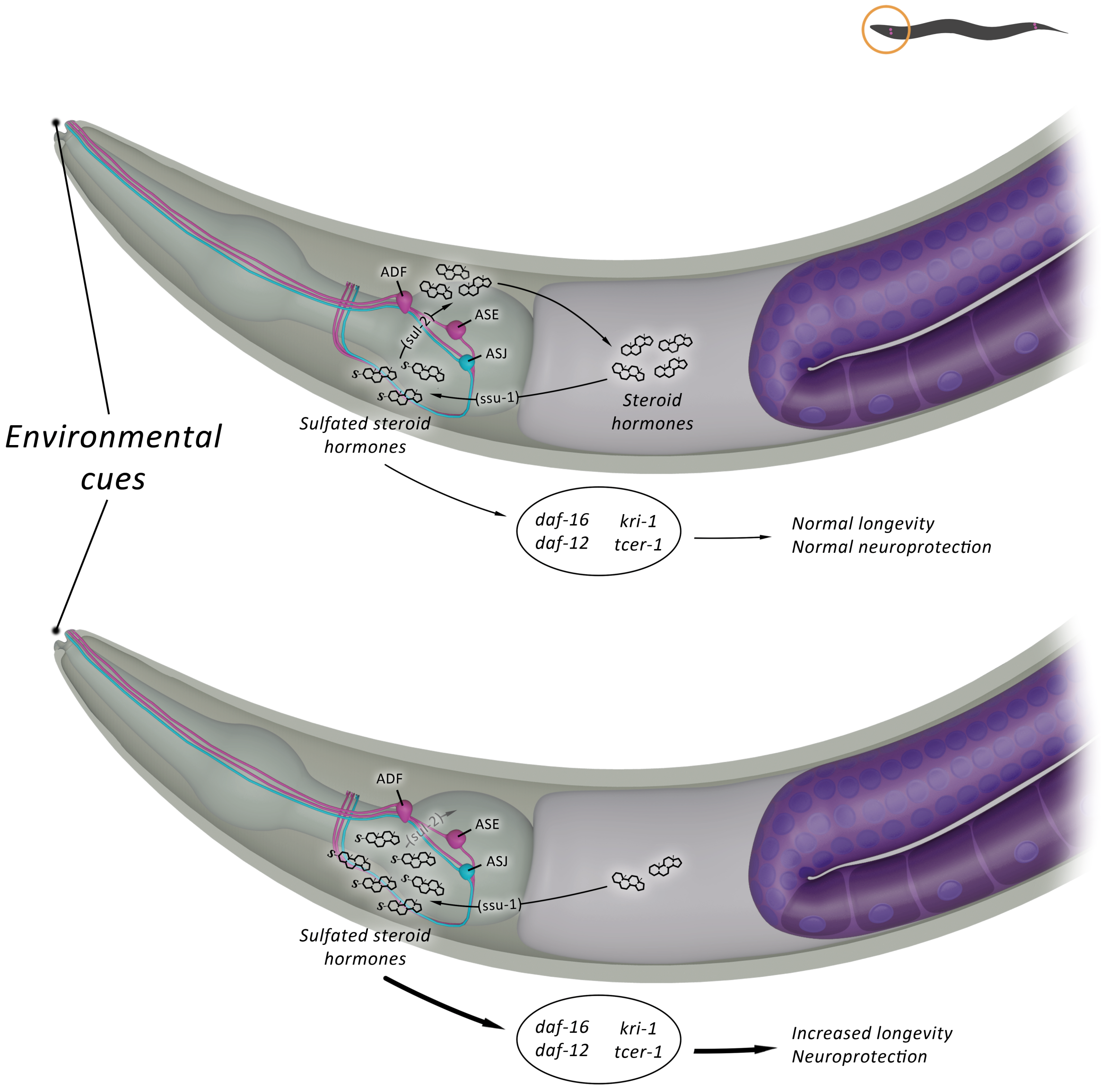

Supplementary Fig. 10 Model of regulation of longevity by SUL-2. The sulfatase SUL-2 and the sulfotransferase (probably *ssu-1*) regulates the level of sulfated steroid hormones. High level of sulfated steroid hormones provoke an increase of longevity, which depend on the same factors to the longevity generated by germline reduction (*daf-16*, *daf-12*, *kri-1*, *tcer-1*). The fact that both sulfatase and sulfotransferase are expressed in sensory neurons suggests a coordination of the sulfated state of the hormones with environmental signals.

Sup.Table 1 | Molecular formula of hormones deteced and % of each specie in wild type and *sul-2(gk187)* for three independent experiments.

Exp:1			**wt**		***sul-2(gk187)***	
**Molecular formula**	**Putative hormones**	**R. T.**	**Area**	**%**	**Area**	**%**
C 21 H 25 D 5 O 4	INTERNAL STANDARD 11-Deoxycortisol-2-2-4-6-6-d5	13,4	592180			
**C 19 H 30 O 6 S 1**	11ceto-3b5a-tetrahydrotestosterone (3b5a-THT) SULFATE	14,3			677647	**38,72**
**C 19 H 28 O 5 S 1**	Dehydroepiandrosterone sulfate (DHEA-S)<br>Testosterone sulfate (T-S)<br>Epitestosterone sulfate	14,4	8455	**0,16**		
C 19 H 30 O 2	Etiocholanolone<br>5a-dihydrotestosterone (5a-DHT)<br>Androsterone<br>Epiandrosterone	30	201986	3,87		
C 21 H 32 O 2	Pregnenolone (PREG)<br>20a-dihydroprogesterone (20a-DH PROG)<br>20b-dihydroprogesterone (20b-DH PROG)<br>3a-dihydroprogesterone (3a-DH PROG)	31,5	955425	18,31	356762	20,38
C 19 H 32 O 2	3a5a-tetrahydrotestosterone (3a5a-THT)<br>3b5a-tetrahydrotestosterone (3b5a-THT)	31,9	664476	12,73	138758	7,93
C 24 H 34 D 4 O 4	INTERNAL STANDARD DEHP_d4	32,7	579652		800004	
C 21 H 34 O 2	3b5a-tetrahydroprogesterone (3b5a-TH PROG)<br>3a5a-tetrahydroprogesterone (3a5a-TH PROG)<br>3a5b-tetrahydroprogesterone (3a5b-TH PROG)<br>20a-dihydropregnenolone (20a-DH PREG)	33,4	2612563	50,07	576976	32,97
C 21 H 34 O 2	Allopregnanolone<br>Epi-allopregnanolone<br>Epipregnanolone<br>5a20b-tetrahydroprogesterone (5a20b-TH PROG)	38,2	775270	14,86		
Exp:2						
C 21 H 25 D 5 O 4	INTERNAL STANDARD 11-Deoxycortisol-2-2-4-6-6-d5	13,3			406742	
C 21 H 25 D 5 O 4	INTERNAL STANDARD 11-Deoxycortisol-2-2-4-6-6-d5	13,4	505721			
**C 19 H 28 O 5 S 1**	Dehydroepiandrosterone sulfate (DHEA-S)<br>Testosterone sulfate (T-S)<br>Epitestosterone sulfate	14,3			7073	**8,31**
**C 19 H 26 O 5 S 1**	D4-androstene 3-17-dione sulfate (ADIONE-S)	19,3	3028	**1,62**		
C 21 H 27 D 4 Na 1 O 5 S 1	INTERNAL STANDARD SODIADO Sodium pregnenolone-17A	26,2			7637	
C 21 H 30 O 2	Progesterone (PROG)	30,9	15475	8,27		
C 21 H 32 O 2	Pregnenolone (PREG)<br>20a-dihydroprogesterone (20a-DH PROG)<br>20b-dihydroprogesterone (20b-DH PROG)<br>3a-dihydroprogesterone (3a-DH PROG)	31,3			64766	76,12
C 21 H 32 O 2	Pregnenolone (PREG)<br>20a-dihydroprogesterone (20a-DH PROG)<br>20b-dihydroprogesterone (20b-DH PROG)<br>3a-dihydroprogesterone (3a-DH PROG)	31,5	120538	64,45		
C 21 H 34 O 2	3a5a-tetrahydroprogesterone (3a5a-TH PROG)<br>3a5b-tetrahydroprogesterone (3a5b-TH PROG)<br>3b5a-tetrahydroprogesterone (3b5a-TH PROG)<br>5a20b-tetrahydroprogesterone (5a20b-TH PROG)	33,2			13250	15,57
C 21 H 34 O 2	3a5a-tetrahydroprogesterone (3a5a-TH PROG)<br>3a5b-tetrahydroprogesterone (3a5b-TH PROG)<br>3b5a-tetrahydroprogesterone (3b5a-TH PROG)<br>20a-dihydropregnenolone (20a-DH PREG)	33,4	32205	17,22		
C 21 H 34 O 2	3a5a-tetrahydroprogesterone (3a5a-TH PROG)<br>3a5b-tetrahydroprogesterone (3a5b-TH PROG)<br>3b5a-tetrahydroprogesterone (3b5a-TH PROG)<br>20a-dihydropregnenolone (20a-DH PREG)	33,8	15790	8,44		
Exp:3						
C 21 H 25 D 5 O 4	INTERNAL STANDARD 11-Deoxycortisol-2-2-4-6-6-d5	13,4	526988		562553	
**C 19 H 30 O 6 S 1**	11ceto-3b5a-tetrahydrotestosterone (3b5a-THT) SULFATE	14,3			982285	**71,78**
**C 19 H 28 O 5 S 1**	Dehydroepiandrosterone sulfate (DHEA-S)<br>Testosterone sulfate (T-S)<br>Epitestosterone sulfate	25			5470	**0,40**
C 21 H 32 O 2	3a-dihydroprogesterone (3a-DH PROG)<br>20b-dihydroprogesterone (20b-DH PROG)<br>20a-dihydroprogesterone (20a-DH PROG)<br>Pregnenolone (PREG)	31,5	315036	63,88	289393	21,15
C 19 H 32 O 2	3a5a-tetrahydrotestosterone (3a5a-THT)<br>3b5a-tetrahydrotestosterone (3b5a-THT)	31,9			13554	0,99
C 19 H 32 O 2	3a5a-tetrahydrotestosterone (3a5a-THT)<br>3b5a-tetrahydrotestosterone (3b5a-THT)	32	20856	4,23		
C 24 H 34 D 4 O 4	INTERNAL STANDARD DEHP_d4	32,8	1289649		996419	
C 21 H 34 O 2	3b5a-tetrahydroprogesterone (3b5a-TH PROG)<br>20a-dihydropregnenolone (20a-DH PREG)<br>3a5b-tetrahydroprogesterone (3a5b-TH PROG)<br>3a5a-tetrahydroprogesterone (3a5a-TH PROG)	33,4	95830	19,43	77811	5,69
C 21 H 34 O 2	3b5a-tetrahydroprogesterone (3b5a-TH PROG)<br>20a-dihydropregnenolone (20a-DH PREG)<br>3a5b-tetrahydroprogesterone (3a5b-TH PROG)<br>3a5a-tetrahydroprogesterone (3a5a-TH PROG)	33,7	61473	12,46		

<

R. T. Retention Time

The compounds represented are those whose molecular formula are identified with an error of < 5ppm and mSigma<50. A maximum of four putative hormones with this formula are indicated in the second column, in blue are those that their assigned retention time (R.T.) are coincident with the R. T. observed in the experiment. In green are those that also the qualifier ions coincide with the putative hormone. The sulfated species and the percentage are indicated in bold. The list of hormones used for the screening are in SupplementaryTable 2.

Sup.Table 2 | Molecular formula of hormones detected and % of each specie in *glp-1(e2141)* for two independent experiments.

			Exp 1		Exp 2	
**Molecular formula**	**Putative hormones**	**R. T.**	**Area**	**%**	**Area**	**%**
C 21 H 25 D 5 O 4	INTERNAL STANDARD 11-Deoxycortisol-2-2-4-6-6-d5	13,4	573486		476213	
C 21 H 28 O 3	11-ceto-progesterone (11ceto PROG)	24,8			9522	6,95
C 19 H 30 O 2	Etiocholanolone<br>5a-dihydrotestosterone (5a-DHT)<br>Androsterone<br>Epiandrosterone	30	164701	4,28		
C 21 H 32 O 2	Pregnenolone (PREG)<br>20a-dihydroprogesterone (20a-DH PROG)<br>20b-dihydroprogesterone (20b-DH PROG)<br>3a-dihydroprogesterone (3a-DH PROG)	31,5	787221	20,46	68365	49,89
C 19 H 32 O 2	3a5a-tetrahydrotestosterone (3a5a-THT)<br>3b5a-tetrahydrotestosterone (3b5a-THT)	31,9	623144	16,20		
C 24 H 34 D 4 O 4	INTERNAL STANDARD DEHP_d4	32,7	734542		357465	
C 21 H 34 O 2	3b5a-tetrahydroprogesterone (3b5a-TH PROG)<br>3a5a-tetrahydroprogesterone (3a5a-TH PROG)<br>3a5b-tetrahydroprogesterone (3a5b-TH PROG)<br>20a-dihydropregnenolone (20a-DH PREG)	33,4	2272308	59,06	24545	17,91
C 21 H 34 O 2	3b5a-tetrahydroprogesterone (3b5a-TH PROG)<br>3a5a-tetrahydroprogesterone (3a5a-TH PROG)<br>3a5b-tetrahydroprogesterone (3a5b-TH PROG)<br>20a-dihydropregnenolone (20a-DH PREG)	33,7			19614	14,31
C 21 H 34 O 2	3b5a-tetrahydroprogesterone (3b5a-TH PROG)<br>3a5a-tetrahydroprogesterone (3a5a-TH PROG)<br>3a5b-tetrahydroprogesterone (3a5b-TH PROG)<br>20a-dihydropregnenolone (20a-DH PREG)	38,3			14980	10,93

R. T. Retention Time

The compounds represented are those whose molecular formula are identified with an error of < 5ppm and mSigma<50. A maximum of four putative hormones with this formula are indicated in the second column, in blue are those that their assigned retention time (R.T.) are coincident with the R. T. observed in the experiment. In green are those that also the qualifier ions coincide with the putative hormone. The sulfated species and the percentage are indicated in bold. The list of hormones used for the screening are in SupplementaryTable 2.

Supplementary Dataset 3. Sequence and details of primers

Primers from mapping pv17 allele					
Chromosome	Allele	Primer sequence (forward)	Primer sequence (reverse)	Restriction enzyme	Ref.
I	pkP1097	atgcaggcattcgacgaatc	gtcgaaaaagcgcacaattc	DraI	Wicks et al. 2001
II	pkP2107	tccacactatttccctcgtg	gagcaatcaagaaccggatc	DraI	Wicks et al. 2001
III	pkP3076	cattaggaagtgatgcaagtgg	tggatttgagaggtgtccatag	AvaII	Wicks et al. 2001
IV	pkP4066	ccaaacaacctacagaaaatgc	aagatattcatgcgtcgtagtg	DraI	Wicks et al. 2001
V	pkP5076	cggaaaattgcgactgtc	attaggactgcttggcttcc	DraI	Wicks et al. 2001
V	pkP5097	gtgctaattccagaaatgatcc	tagtgttcatagcatcccattg	DraI	Wicks et al. 2001
V	pkP5114	agagcgagatcttcacagcaag	aaaactggcgttgacgaaatc	AluI	wormbase
V	ce5-168	cgaagggacgaaaatattgaa	cgacagaaaaattacatacctgtctt	-	This project
V	D1014[1]	aaatatttttctgaccacacacg	ggcttctgtgcatccatttt	-	This project
V	pkP5062	gagagttcactgactatggc	cgaggaatgagcagttcagtag	AluI	wormbase
V	pkP5085	tggttgggaaaaaactcgac	atcgcgaaatttcgtggttc	SacI	This project
V	pkP5129	ggcggaaagcaatttctatc	agctgcaaccaacactgctc	DraI	This project
V	pkP5082	caggcatattacatgggatagg	caatctcacctccattctgtg	DraI	Wicks et al. 2001
X	pkP6110	tttcttgacacctccggtag	ctcactctggtctttttccg	EcoRI	Wicks et al. 2001

sul-2 primers			
Primer name	Primer sequence (forward)	Primer sequence (reverse)	Annotations
exon1.sul2.ext	AGTGATTCTTCGTGGGCATT	ACGATTTTCAAACGGCAAAG	pv17 sequencing (external primers)
exon1.sul2.int	GCTTCTTTCTCGGCTTCCTC	TTGTGTTTCCGAGACATTTCA	pv17 sequencing (internal primers)
all_sul2	GGCCTTGTTTTCAGACCAGT	TTTTTGGACGATGAC	pv17 complementation (Prom+Gene)
curated_sul2	TCCACAAGTCCACTCAACACA	TCTCCCATCCTAATAGCCATC	For curated sequencing of sul-2
Psul-2	AAAGATTTTTAACTGCCGTTTTTC	*GATCTGAAAGATTATGAATGAAATCAA*	Amplified 1772 pb upstream sul-2 gene
gk187_pv17	CTTCGTGGGCATTAGTATGTAGT	TGTTTTCATTAATTCCAAGATGC	To follow both sul-2 alleles. 50ºC, 1'. For pv17 (Dig with BcgI)
3UTR_sul-2+BamHI	AT**GGATCC**GAATTTCTAAAATTC	TA**GGATCC**ATTTTGTAAATTAGCAC	For sul-2 3'UTR clonning

Primers to follow other mutants			
Primer name	Primer sequence (forward)	Primer sequence (reverse)	Annotations
e979	AAAATTTCCGGCAAATCCAT	TTTGCCGATTTTCCGTGTAT	To follow daf-2(e979)
m20	TTCTTCAGGAGAAATGCGTTA	CATATATTGCAACTCCTGGATGAT	To follow daf-12(m20). 58ºC, 1'+ Dig con ApoI
ok1251	CAAAGTGCTCCGCTGAAGAT	CGCCAGTGAGGAGAGTGTTT	To follow kri-1(ok1251)
gk151	TGTGTCTCGTTTTTGGTTCG	CGCTCCAAATCCTGATAAGC	To follow sul-1(gk151)
tm1452	GCAAACCCGCCTCACTAAAT	ATTCGCAGGTAGAGGTCGTG	To follow tcer-1(tm1452)
tm1011	CTTTCCGGAGCAGTGACATT	CAGGTATGCACCAAATCTGC	To follow nhr-80(tm1011)
nr2041	TCCAAAACACAGTGGCCTCA	GAAGTTGTCGCATTGGCCAG	To follow nhr-49(nr2041)
tm6179	GCATATCAGCAGGTGGGAAT	CATTTCCTATGTAGCCGGAC	To follow sul-3(tm6179)
mu86	GTAGACGGTGACCATCTAGAG	AGCTCACACACACACATGCG<br>GAAACACGAGACGACGATCC	To follow mu86. We combined 3 primers
