# Supplementary material for: Steroid hormones sulfatase inactivation extends lifespan and ameliorates age-related diseases
Source: Nat Commun. 2021 Jan 4;12:49. doi: 10.1038/s41467-020-20269-y (PMC7782729; doi:10.1038/s41467-020-20269-y)
Supplement: Supplementary file 3 — Description of Additional Supplementary Files [file 41467_2020_20269_MOESM3_ESM.pdf]

Description of Additional Supplementary Files

File Name: Supplementary Data File 1: Lifespan analyses

File Name: Supplementary Data File 2: Data base used for hormone screening

File Name: Supplementary Movie 1: Thrashing assay in a wild type background.
Representative *C. elegans* model for Parkinson in a wild type background, at day 7.

File Name: Supplementary Movie 2: Thrashing assay in a *sul-2* mutant background.
Representative *C. elegans* model for Parkinson in a *sul-2(gk187)* background, at day 7. Notice the improved movement compared to control (Supplementary video 1).

File Name: Supplementary Movie 3: Thrashing assay. Parkinson model treated with vehicle.
Non-treated *C. elegans* model for Parkinson. Representative individual of the population, at day 10.

File Name: Supplementary Movie 4: Thrashing assay. Parkinson model treated with STX64.
Representative individual of the population, at day 10. Notice the improved movement compared to control (Supplementary video 3).
