# Supplementary material for: Steroid hormones sulfatase inactivation extends lifespan and ameliorates age-related diseases
Source: Nat Commun. 2021 Jan 4;12:49. doi: 10.1038/s41467-020-20269-y (PMC7782729; doi:10.1038/s41467-020-20269-y)
Supplement: Supplementary file 10 — Reporting Summary [file 41467_2020_20269_MOESM10_ESM.pdf]

nature research

Corresponding author(s): Manuel J. Muñoz

Last updated by author(s): 2020/09/21

Reporting Summary

Nature Research wishes to improve the reproducibility of the work that we publish. This form provides structure for consistency and transparency in reporting. For further information on Nature Research policies, see our Editorial Policies and the Editorial Policy Checklist.

Statistics

For all statistical analyses, confirm that the following items are present in the figure legend, table legend, main text, or Methods section.

n/a	Confirmed	
☐	☒	The exact sample size (*n*) for each experimental group/condition, given as a discrete number and unit of measurement
☐	☒	A statement on whether measurements were taken from distinct samples or whether the same sample was measured repeatedly
☐	☒	The statistical test(s) used AND whether they are one- or two-sided<br>*Only common tests should be described solely by name; describe more complex techniques in the Methods section.*
☐	☒	A description of all covariates tested
☐	☒	A description of any assumptions or corrections, such as tests of normality and adjustment for multiple comparisons
☐	☒	A full description of the statistical parameters including central tendency (e.g. means) or other basic estimates (e.g. regression coefficient) AND variation (e.g. standard deviation) or associated estimates of uncertainty (e.g. confidence intervals)
☐	☒	For null hypothesis testing, the test statistic (e.g. *F*, *t*, *r*) with confidence intervals, effect sizes, degrees of freedom and *P* value noted<br>*Give P values as exact values whenever suitable.*
☒	☐	For Bayesian analysis, information on the choice of priors and Markov chain Monte Carlo settings
☒	☐	For hierarchical and complex designs, identification of the appropriate level for tests and full reporting of outcomes
☒	☐	Estimates of effect sizes (e.g. Cohen's *d*, Pearson's *r*), indicating how they were calculated

Our web collection on statistics for biologists contains articles on many of the points above.

Software and code

Policy information about availability of computer code

Data collection	*Provide a description of all commercial, open source and custom code used to collect the data in this study, specifying the version used OR state that no software was used.*
Data analysis	FigTree v.1.4.3, Graph Prism 5(Version 5.0a), Microsoft Excel 2016, Bruker Compass DataAnalysis 4.1, TargetAnalysisTM 1.2, Image-J. 1.51k were used as indicated in material and method section

For manuscripts utilizing custom algorithms or software that are central to the research but not yet described in published literature, software must be made available to editors and reviewers. We strongly encourage code deposition in a community repository (e.g. GitHub). See the Nature Research guidelines for submitting code & software for further information.

Data

Policy information about availability of data

All manuscripts must include a data availability statement. This statement should provide the following information, where applicable:

- Accession codes, unique identifiers, or web links for publicly available datasets
- A list of figures that have associated raw data
- A description of any restrictions on data availability

Authors can confirm that all relevant data are included in the paper and/ or its supplementary information files

Field-specific reporting

Please select the one below that is the best fit for your research. If you are not sure, read the appropriate sections before making your selection.

- [x] Life sciences
- [ ] Behavioural & social sciences
- [ ] Ecological, evolutionary & environmental sciences

For a reference copy of the document with all sections, see nature.com/documents/nr-reporting-summary-flat.pdf

Life sciences study design

All studies must disclose on these points even when the disclosure is negative.

Sample size	Sample size and statistical test were chosen based on previous studies that used the same or similar methods, except for steroid purification that each sample contained aprox. 100.000 worms.<br><br>For longevity Hsin H and Kenyon. C. Nature 399, 362-366 (1999).<br><br>For brood size Houthoofd K. et al. Aging Cell 4, 85-79 (2005)<br><br>For Fluorescence microscopy Monje et al. Aging Cell 10, 1921-1031 (2011)<br><br>For trashing assays van Ham TJ, et al. Cell 142, 601-612 (2010).<br><br>For Paralysis assay McColl G, et al. Mol Neurodegener 7, 57 (2012).<br><br>For Dauer analysis Monje et al. Aging Cell 10, 1921-1031 (2011)<br><br>For meassurement of health span (pumping and body bends) Huang et al. PNAS. 101:8084-8089 (2004)<br><br>For Chemiotaxis Bargmann et a. Neuron 7, 129-142 (1991)<br><br>For experiements using mice: Bachiller et al. Barin Behav Immun. 64:65-70 (2017) and Suárez-Pereira I, et al. Hippocampus. 1:51-61. (2015)<br><br>Please see the figure legends for the precise number of replicates
Data exclusions	No data were excluded from this manunscript.
Replication	All data in the manuscript was obtained with at least two biological replicates.
Randomization	For all experiment animals were randomly selected
Blinding	Where feasible the experimenter was blinded to animal genotype.

Reporting for specific materials, systems and methods

We require information from authors about some types of materials, experimental systems and methods used in many studies. Here, indicate whether each material, system or method listed is relevant to your study. If you are not sure if a list item applies to your research, read the appropriate section before selecting a response.

Materials & experimental systems

n/a	Involved in the study	
[ ]	[x]	Antibodies
[x]	[ ]	Eukaryotic cell lines
[x]	[ ]	Palaeontology and archaeology
[ ]	[x]	Animals and other organisms
[x]	[ ]	Human research participants
[x]	[ ]	Clinical data
[x]	[ ]	Dual use research of concern

Methods

n/a	Involved in the study	
[x]	[ ]	ChIP-seq
[x]	[ ]	Flow cytometry
[x]	[ ]	MRI-based neuroimaging

Antibodies

Antibodies used	Antibody against βA : Clone BAM-10, Sigma Aldrich Ref: A3981

Validation	Antibodies are validated by manufacturer and in this study.

Animals and other organisms

Policy information about studies involving animals; ARRIVE guidelines recommended for reporting animal research

Laboratory animals	Caenorhabditis elegans, mouse. Housing conditions for the mice were 12 light/12 dark cycle, Temperatures of ~21-23°C with 40-60% humidity. The mice had access to food and drink ad libitum.
Wild animals	No wild animals were used in this study
Field-collected samples	This study did not involve samples collected from the field.
Ethics oversight	Experiments using mice were performed under protocols approved by the Universidad Pablo de Olavide Ethical Committee (Sevilla, Spain; protocols 24/04/2018/043) in accordance with Spanish Royal Decree 53/2013, European Directive 2010/63/EU, and other relevant guidelines.

Note that full information on the approval of the study protocol must also be provided in the manuscript.
